# Identification of JL1037 as a novel, specific, reversible lysine-specific demethylase 1 inhibitor that induce apoptosis and autophagy of AML cells

**DOI:** 10.18632/oncotarget.16650

**Published:** 2017-03-29

**Authors:** Shuang Liu, Wenting Lu, Shouyun Li, Saisai Li, Jia Liu, Yuanyuan Xing, Shuzu Zhang, Joe Zhongxiang Zhou, Haiyan Xing, Yingxi Xu, Qing Rao, Chengjun Deng, Min Wang, Jianxiang Wang

**Affiliations:** ^1^ State Key Laboratory of Experimental Hematology, Institute of Hematology and Blood Diseases Hospital, Chinese Academy of Medical Sciences and Peking Union Medical College, Tianjin 300020, China; ^2^ Fujian Jinler Pharmaceuticals, Jiangle County, Fujian 353300, China

**Keywords:** LSD1 inhibitor, leukemia, proliferation inhibition, apoptosis, autophagy

## Abstract

Lysine-specific demethylase 1 (LSD1) has been recognized as a potential therapeutic target for acute myeloid leukemia (AML). Herein, we identified a novel LSD1 inhibitor, JL1037, via Computer Aided Drug Design technology. JL1037 is a potent, selective and reversible LSD1 inhibitor with IC50s of 0.1 μM and >1.5 μM for LSD1 and monoamine oxidases A/B (MAO-A/B), respectively. Treatment of THP-1 and Kasumi-1 cell lines with JL1037 resulted in dose dependent accumulation of H3K4me1 and H3K4me2, the major substrates of LSD1, as well as inhibition of cell proliferation, blockade of cell cycle and induction of apoptosis. Further investigations demonstrated that JL1037 could upregulate cell cycle-related proteins P21, P57, pro-apoptotic protein Bax and downregulate anti-apoptosis proteins Bcl-2 and Bcl-XL. JL1037 appeared to activate autophage response in AML cell lines as well as primary cells from AML patients by increasing LC3-II expression and the formation of autophagosomes and autolysosomes in cytoplasm. Co-treatment with autophagy inhibitor chloroquine (CQ) enhanced JL1037-induced cell apoptosis. Moreover, daily intravenous administration of JL1037 tended to reduce tumor burden and prolong the survival of t(8;21) leukemia mice. In conclusion, JL1037 exhibited potent anti-leukemia effect and could be a potential therapeutic agent for AML treatment.

## INTRODUCTION

Epigenetic abnormalities are in close relationship with the initiation and progression of leukemia [1, 2]. Since epigenetic abnormalities are generally reversible by nature versus gene mutations, epigenetic targets have great potentials for developing novel and more effective therapeutic drugs for leukemia treatment. Abnormality in histone lysine methylation modifications, regulated by histone methyltransferases and histone lysine demethylases (LSD), is one of the most common epigenetic dysfunctions found in numerous types of cancers including various forms of leukemia [3–7]. LSD1, the first reported histone demethylase [8], participates in many multisubunit complexes and serves as either a transcriptional repress or a transcriptional activator depending on cell context [9–12]. In most cases, LSD1 is a transcriptional repressor suppressing the expression of tumor suppressor genes through demethylating H3K4 in the promoter/enhancer regions, and therefore promotes tumorigenesis.

More and more investigators have reported that LSD1 is highly expressed and closely correlated with poor prognosis in various kinds of malignancies [13–16]. Harris and his colleagues had demonstrated that LSD1 was preferentially expressed in leukemia stem cell (LSC)-enriched population and functioned to maintain LSC potential of AML cells. LSD1 knockdown or pharmacological inhibition reduces the colony-forming cell (CFC) frequencies of MLL-AF9 AML cells and induces cell differentiation [17]. The finding provides further mechanism evidence for developing small-molecule LSD1 inhibitors to treat AML and other types of leukemia. Over the past few years, a large number of irreversible inhibitors of LSD1 have been developed by major pharmaceutical companies and research institutes [18–20]. Among them, ORY-1001 and GSK2879552, two TCP derivatives, developed by Oryzon Genomics and GSK, respectively, have been in phase I clinical trials for treatment of AML and small cell lung cancer, respectively. Although the above irreversible LSD1 inhibitors exhibit potent and long-lasting biological effects, they may also produce endurable side-effects due to extensive LSD1 inactivation. Effective reversible LSD1 inhibitors may be more desirable in terms of the control of the target suppression and may alleviate some of the side-effects caused by irreversible inhibitors, such as bone marrow suppression [17].

In this study, using structure-based virtual screening and subsequent compound optimization, we identified and synthesized a novel reversible LSD1 inhibitor coded as JL1037, which specifically inhibited LSD1 in cell-free enzymatic assay with great potency. We further characterized JL1037 in *ex vivo* AML cell systems as well as in a mouse model harboring AML1-ETO translocation to elucidate the role of JL1037 as an effective anti-leukemia agent.

## RESULTS

### LSD1 is highly expressed in AML cells

**Figure 1:**
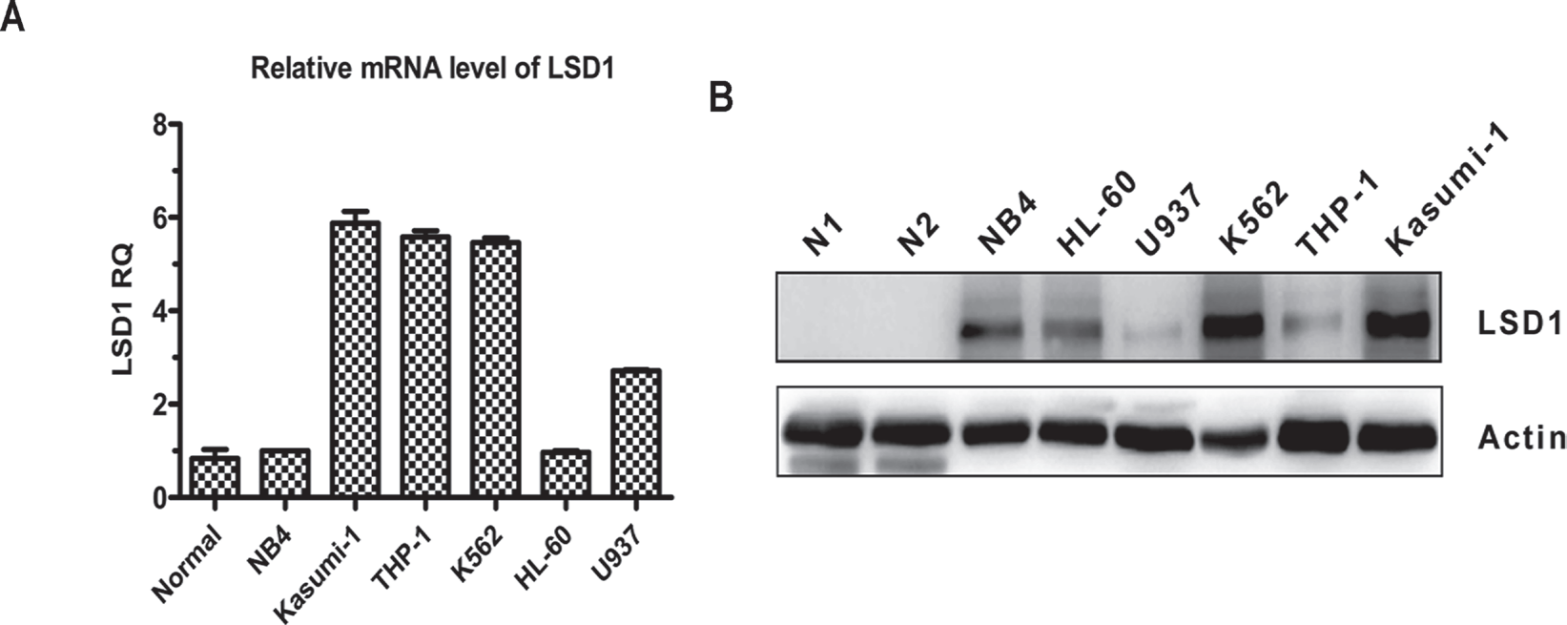
LSD1 expression is elevated in AML cell lines compared with that of normal BMMNCs (**A**) Relative expression of LSD1 mRNA was measured by qRT-PCR in AML cell lines and normal BMMNCs from 5 healthy donors. Data are represented as means ± SD. (**B**) Western blot analysis of LSD1 protein in AML cell lines and normal BMMNCs. β-actin was used as an internal loading control.

To investigate whether LSD1 could be a valid therapeutic target for AML, the expression levels of LSD1 in a variety of AML cell lines were compared with that of normal bone marrow mononuclear cells (BMMNCs) by real-time quantitative PCR (qRT-PCR) and Western blot. The results clearly demonstrated that LSD1 expression at both mRNA and protein level was significantly higher in majority of AML cell lines, especially in Kasumi-1, THP-1 and K562 cells, compared with that of normal BMMNCs, which was hardly detectable (Figure 1A and 1B).

### JL1037 is a novel LSD1 specific inhibitor

**Figure 2:**
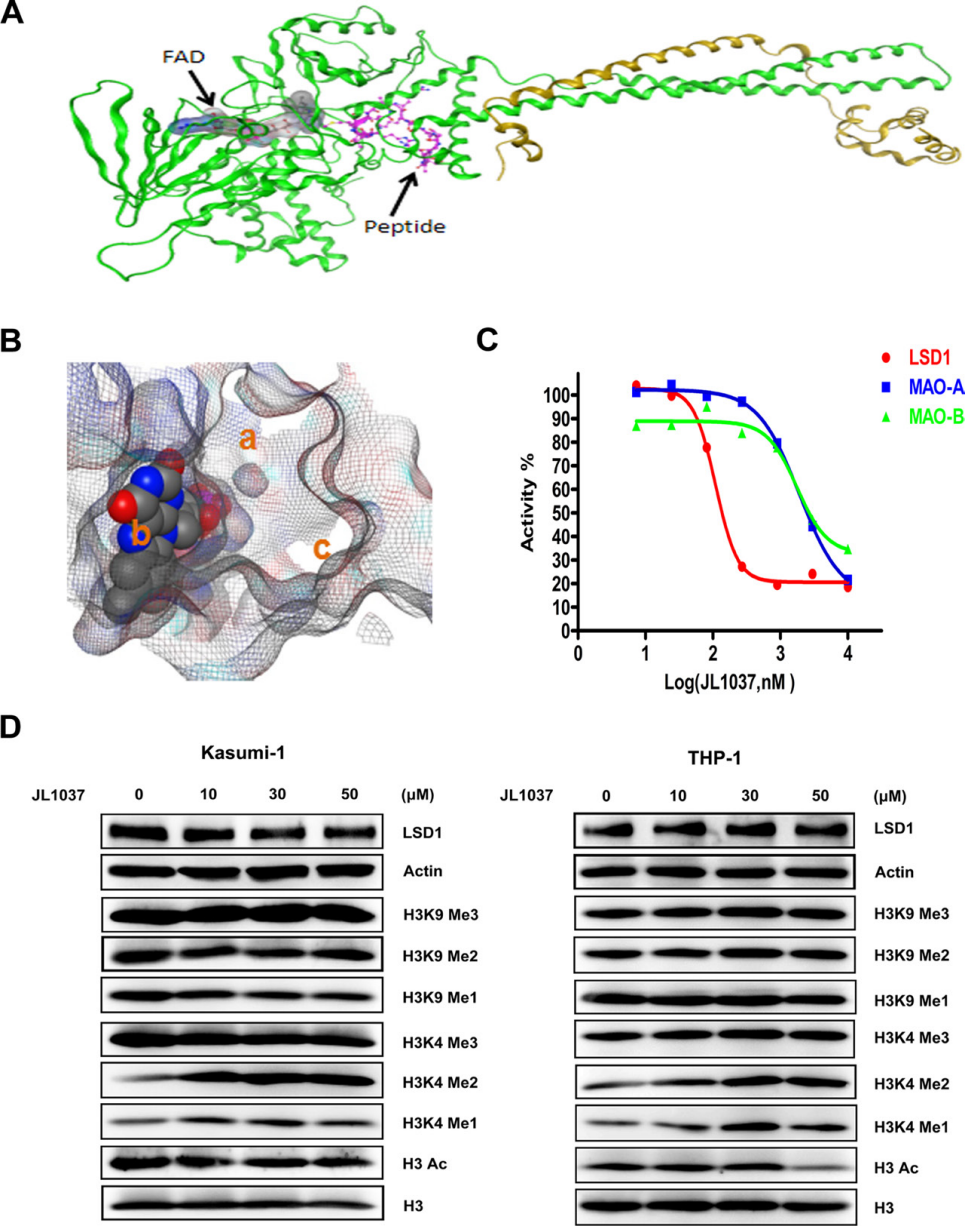
Docking strategy of compound JL1037 and its LSD1 specific inhibitory activity (**A**) Overall structure of LSD1–CoREST–Peptide complex. LSD1 (green), CoREST (orange) are drawn. (**B**) The three sub-pockets (a, b and c) filled by JL1037 in the active site of LSD1. The close packing model represents FAD. (**C**) JL1037 enzymatic inhibitory activity against LSD1, MAO-A and MAO-B. (**D**) Western blot analysis to evaluate the effect of JL1037 on LSD1 expression and modifications of histone H3. THP-1 and Kasumi-1 cells were seeded in 6-well plates at a density of 5 × 10^5^ cells/ml and treated with increasing concentrations of JL1037 for 48 h, then whole cell lysates were analyzed by Western blot with the indicated antibodies. β-actin and total H3 were used as internal loading controls.

Increasing numbers of investigators have demonstrated LSD1 as a potentially promising drug target for AML. Herein, we successfully synthesized a novel LSD1 inhibitor, JL1037, which was originated from computational screening and designed using mainly the DOCK module of the MOE software (CCG, Montreal, Canada). Molecular docking sampled conformations of small molecules in a protein binding site and found which of those shapes fit well both spatially and chemically to the protein binding site. In this study, docking was implemented with the crystal structure of the LSD1 enzyme in complex with CoREST and a substrate-like peptide (PDB ID: 2VID) as shown in Figure 2A. In our docking experiment, JL1037 bound LSD1 well. In close proximity to FAD, JL1037 occupied three important sub-pockets of the active site as shown in Figure 2B. The spatial complementarity played a key role in the JL1037 binding to LSD1. Also in our docking model, JL1037 interacted chemically with LSD1 favorably. JL1037 formed favorable hydrophobic interactions with LSD1 and an important hydrogen bond with the carbonyl oxygen (O4) of FAD. Still more, JL1037 seemed to form favorable charge interactions with LSD1 involving residues Asp553, Asp556, Asp555, and Glu559. Herein, for the sake of the patent protection, the chemical structural formula of JL1037 was not shown.

We next evaluated the *in vitro* inhibitory activity of JL1037 on LSD1 with LSD1 Fluorimetric Drug Discovery kit (# BML-AK 544, Enzo Life Science Inc, USA). JL1037 exhibited good inhibitory potency against LSD1 with IC_50_ value of 110 nM (Figure 2C). We also examined the specificity of JL1037 over other related monoamine oxidases such as MAO-A and MAO-B as the previous LSD1 inhibitors were proved to be strong MAO-A/B inhibitors. We found that the inhibitory effect of JL1037 on LSD1 was 17.45 and 16.09 fold stronger than that on MAO-A and MAO-B, respectively (Table 1), suggesting that JL1037 was a highly specific LSD1 inhibitor. Then, we evaluated JL1037 inhibitory activity against LSD1 at cellular level. Representative AML cell lines THP-1 and Kasumi-1 were treated with increasing doses of JL1037 ranging from 0 μM to 50 μM for 48 hours. Western blot assay was performed to determine the effect of JL1037 on the methylation levels of LSD1 substrates H3K4 and H3K9. In each cell line, JL1037 treatment didn't change the expression level of LSD1, but resulted in detectable increases of H3K4me1 and H3K4me2 (Figure 2D), whereas the levels of acetylated H3, H3K9me1, H3K9me2, H3K4me3 and H3K9me3 remained unchanged. These results suggested that JL1037 was capable of LSD1 inhibition in AML cell lines without affecting the acetylation status of those LSD1 substrates.

**Table 1:** *In vitro* LSD1-, MAO-A and MAO-B inhibitory activities of compound JL1037

IC_50_ (μM)			Selectivity Index	
LSD1	MAO-A	MAO-B	MAO-A/LSD1	MAO-B/LSD1
0.11	1.92	1.77	17.45	16.09

JL1037 enzymatic inhibitory activity against LSD1, MAO-A and MAO-B. The specificity for LSD1 was 17.45 fold over MAO-A and 16.09 fold over MAO-B as shown in the table.

### JL1037 inhibits AML cell proliferation by blocking cell cycles and inducing caspase-3 dependent apoptosis

**Figure 3:**
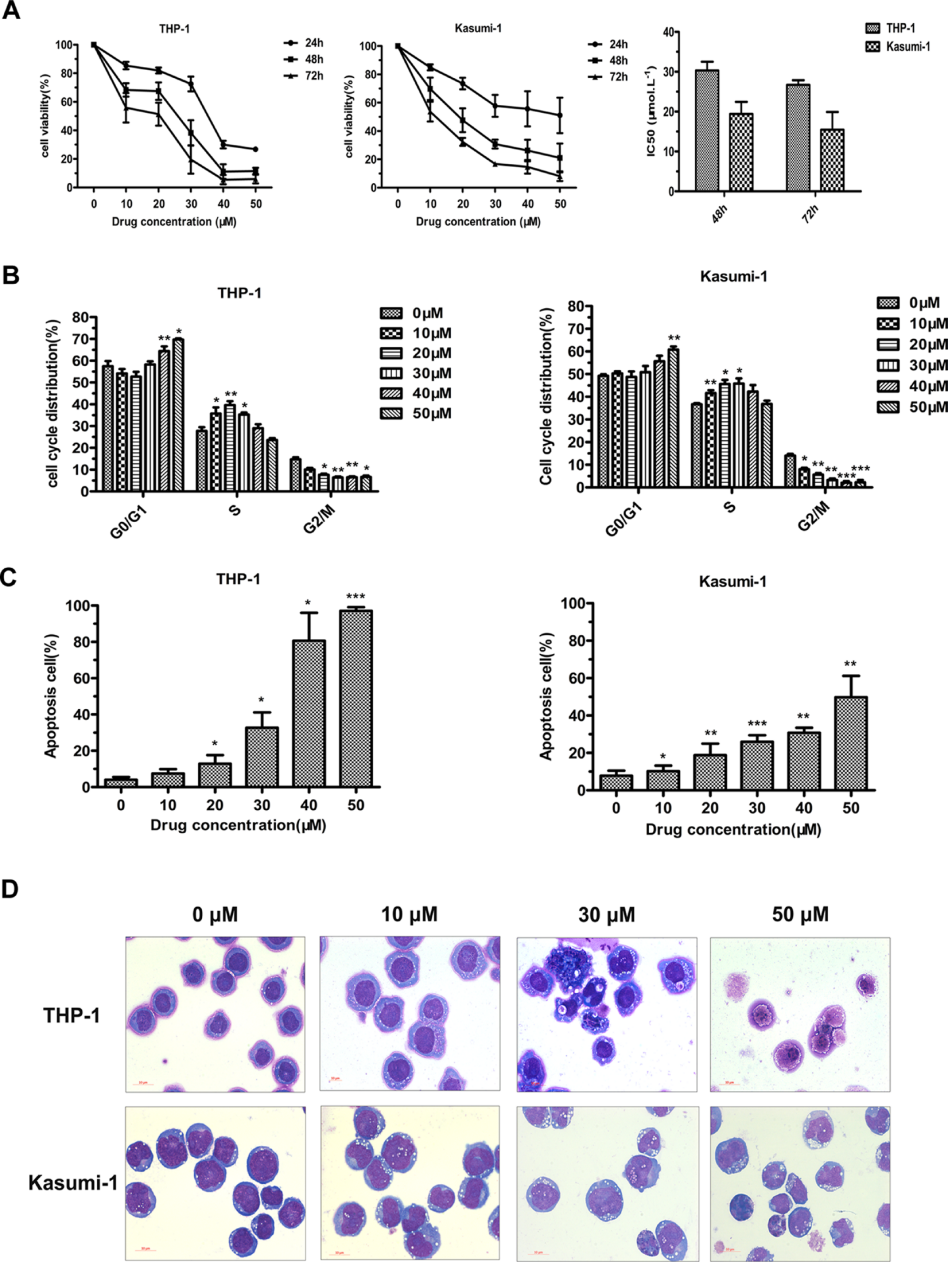
JL1037 inhibits cell proliferation and induces cell cycle arrest and apoptosis in THP-1 and Kasumi-1 cells (**A**) Cell viability analysis. THP-1 and Kasumi-1 cells were seeded in 96-well plates (2 × 10^4^ cells/well), treated with different doses of JL1037, and subjected to the MTS assay at 24 h, 48 h and 72 h. The line-dot curves showed cell viability of THP-1 and Kasumi-1 cells incubated with different concentrations of JL1037 for indicated hours. The bar charts showed IC50 values of JL1037 for each cell lines at 48 h and 72 h. Results were represented as means ± SD from at least three independent experiments. (**B**) Cell cycle analysis.THP-1 and Kasumi-1 cells were seeded in 6-well plates at a density of 5 × 10^5^ cells/ml and treated with increasing doses of JL1037 for 24 h. Then cells were fixed with 70% ethanol overnight at 4°C and analyzed by flow cytometry after propidium iodide staining. The percentage of cell cycle distribution was calculated by the ModFit software. (**C**) Cell apoptosis analysis. THP-1 and Kasumi-1 cells were seeded in 6-well plates at a density of 5 × 10^5^ cells/ml and treated with different concentrations of JL1037 for 48 h. Then apoptotic cells were determined by the flow cytometry using Annexin V/PI staining. Results were represented as means ± SD from three independent experiments. (**D**) Morphological assessment of THP-1 and Kasumi-1 cells treated with different concentrations of JL1037 for 48 h. Cells were stained by Wright-Giemsa staining and observed by oil microscopy after treatment (magnification was 60×).

We next explored the biological impacts of JL1037 on AML cells. MTS assay was performed to evaluate the anti-proliferative activity of JL1037. JL1037 strongly inhibited the growth of THP-1 and Kasumi-1 cells in a time and dose dependent manner. The IC50 values of 48 hours were (30.3 ± 2.22) μM and (19.42 ± 3.02) μM, respectively (Figure 3A). We further tested whether JL1037 inhibited cell proliferation by blocking cell cycles or inducing apoptosis. Cell cycle assay was performed with propidium iodide (PI) staining and the result showed that a 24-hour treatment of JL1037 arrested cell cycle at S phase when used at lower doses (10 ~ 30) μM, however, when the doses increased to (40 ~ 50) μM, the cell cycle would be blocked at G0/G1 phase (Figure 3B). Apoptosis analysis demonstrated that JL1037 induced cell apoptosis in a dose-dependent manner ranging from 10% ~ 90% in THP-1 cells and 10% ~ 60% in Kasumi-1 cells when treated with (10 ~ 50) μM for 48 hours (Figure 3C). Morphology analysis with Wright-Giemsa staining displayed typical apoptotic characteristics of the treated AML cells such as karyopyknosis, reduced ratio of nucleus to cytoplasm, nuclear fragmentation with intact cell membrane and vacuolar degeneration (Figure 3D).

**Figure 4:**
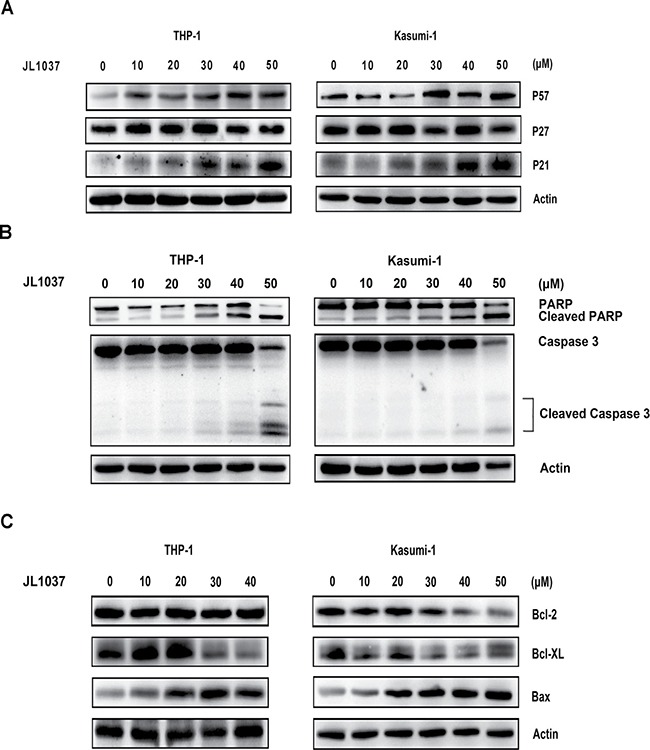
Molecular mechanisms of cell cycle arrest and apoptosis induced by JL1037 (**A**) Western blot analysis to detect the effect of JL1037 on the expression of cell cycle related-protein P57, P27 and P21 in THP-1 and Kasumi-1 cells (5 × 10^5^ cells/ml) exposed to different concentrations of JL1037 for 48 h. β-actin was used as an internal loading control. (**B**) Western blot analysis of cleaved PARP and cleaved caspase-3 in THP-1 and Kasumi-1 cells (5 × 10^5^ cells/ml) incubated with different concentrations of JL1037 for 48 h. β-actin was used as an internal loading control. (**C**) Western blot analysis of BCL-2 family members in THP-1 and Kasumi-1 cells. Cells (5 × 10^5^ cells/ml) were treated with increasing doses of JL1037 for 48 h and harvested for western blot assay using antibodies specifically against Bcl-2, Bcl-xL and Bax. β-actin was used as an internal loading control.

The mechanism underlying the cell cycle arrest and apoptosis induced by JL1037 was explored. Cell cycle-related proteins P21, P27 and P57 were examined by Western blot after 24-hour treatment of the cell lines with JL1037. As shown in Figure 4A, P21 and P57 were significantly up-regulated while P27 remained unchanged. Apoptosis markers such as cleaved caspase-3 and cleaved PARP were also examined after treatment with JL1037 for 48h. Both markers were elevated as the concentrations of JL1037 increased and became more evident when concentration increased to 30 μM or higher (Figure 4B). Bcl-2 family members, the key components of mitochondrial pathway were further investigated. The results demonstrated that the pro-apoptotic protein Bax increased in both cell lines accompanied by the decrease of anti-apoptosis protein Bcl-XL. However, Bcl-2 was down-regulated only in Kasumi-1 cells (Figure 4C).

### Inhibition of JL1037 induced cell autophagy enhances apoptosis of AML cells

**Figure 5:**
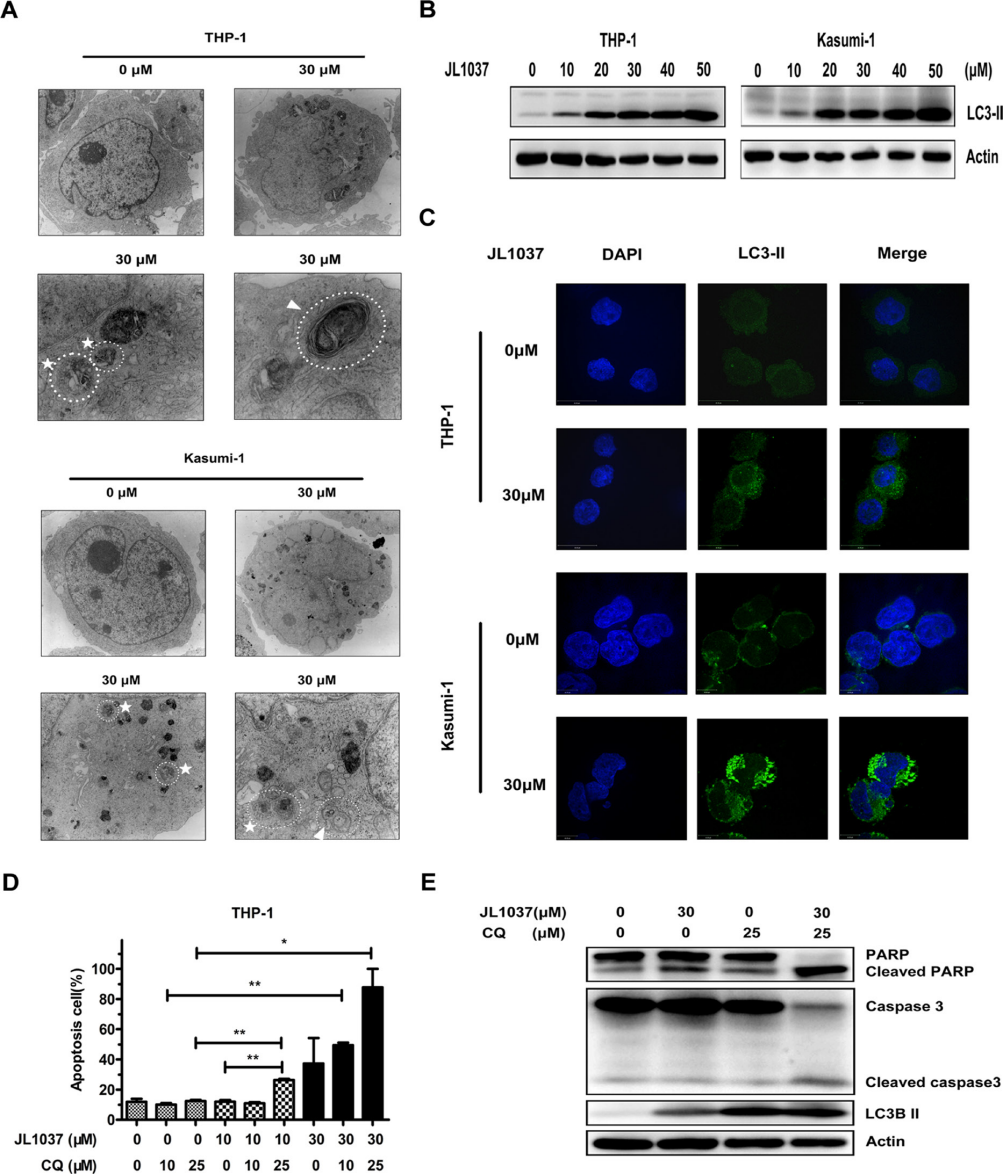
JL1037 induces cell autophagy in THP-1 and Kasumi-1 cells (**A**) THP-1 and Kasumi-1 cells (5 × 10^5^ cells/ml) were incubated with or without JL1037 for 48 h. Then cells were harvested and fixed for transmission electron microscopy (TEM) observation. By TEM, both autophagosomes (represented by arrows) and autolysosomes (represented by asterisks) were clearly observed in the cytoplasm of THP-1 and Kasumi-1 cells in JL1037 treated group, but none of them were detected in untreated group. (**B**) Autophagy-related protein LC3-II was detected in THP-1 and Kasumi-1 cells by Western blot after treated with different concentrations of JL1037 for 48 h. β-actin was used as an internal control. (**C**) Immunofluorescence analysis for LC3-II in THP-1 and Kasumi-1 cells. Cells were exposed to JL1037 (0 μM or 30 μM) for 48 h and then fixed and immunofluorescence stained using specific antibody against LC3-II (green). DAPI (blue) was used for nuclear staining. Bars represent 10 μm. (**D**) THP-1 cells were treated with JL1037 ( 0 μM, 10 μM and 30 μM) in combination with different dosages (0 μM, 10 μM and 25 μM) of autophagy inhibitor chloroquine (CQ) for 48 h, then cells were collected for apoptosis analysis with Annexin V/PI staining by flow cytometry. (**E**) Expression of apoptotic markers cleaved PARP, cleaved caspase-3 and autophagy-related protein LC3-II were assessed by Western blot in THP-1 cells treated with JL1037 (30 μM) in the absence or presence of CQ (25 μM) for 48 h. β-actin was used as an internal loading control.

Autophagy, a kind of non-apoptotic programmed cell death, can be induced by many cytotoxic compounds. In present study, to explore whether autophagy was involved in the process of cell death induced by JL1037, we utilized transmission electron microscopy (TEM) to analyze the ultrastructure of AML cells treated by JL1037. Both autophagosomes and autolysosomes were clearly observed in THP-1 and Kasumi-1 cells after being treated with 30 μM of JL1037 for 48 hours and neither was seen in untreated cells (Figure 5A). Under TEM examination, we also observed features of mitochondrial apoptosis in JL1037 treated cells such as mitochondrial swelling and degeneration, which were consistent with our apoptotic biomarker analyses shown in Figure 4C that JL1037 induced apoptosis through mitochondrial apoptosis pathway (Figure 5A).

The conversion of LC3-I into LC3-II is an essential step in autophagy and the number of LC3-II puncta represents the number of autophagosomes. Western blot assay showed that JL1037 significantly enhanced the expression of LC3-II even at the concentration of 10 μM (Figure 5B). Immunofluorescent staining for LC3-II further revealed that the number of LC3-II puncta in cytoplasm significantly increased in JL1037 treated cells in comparison with the non-treated controls (Figure 5C). Previous studies have demonstrated autophagy could either serve as a pro-apoptotic or an anti-apoptotic factor in tumor cells treated with different anticancer drugs [21–23]. To further explore the relationship between apoptosis and autophagy in AML cells induced by JL1037, THP-1 cells were treated with JL1037 in the presence or absence of a specific autophagy inhibitor chloroquine (CQ). As shown in Figure 5D, the combined treatment with JL1037 and CQ increased more markedly the proportion of apoptotic cells than treatment with JL1037 alone. Also the expression of apoptosis markers such as cleaved PARP and cleaved caspase-3 increased more significantly when JL1037 and CQ were used in combination than used alone (Figure 5E). These results suggested autophagy induced by JL1037 might be an alternative conduit of cell-killing other than apoptosis in the treated AML cells. The combination of JL1037 with an autophagy inhibitor would force more tumor cells to go through apoptosis, therefore, further enhanced the efficacy of LSD1 inhibitors such as JL1037 against leukemia cells.

### JL1037 induces apoptosis and autophagy in primary AML cells

**Figure 6:**
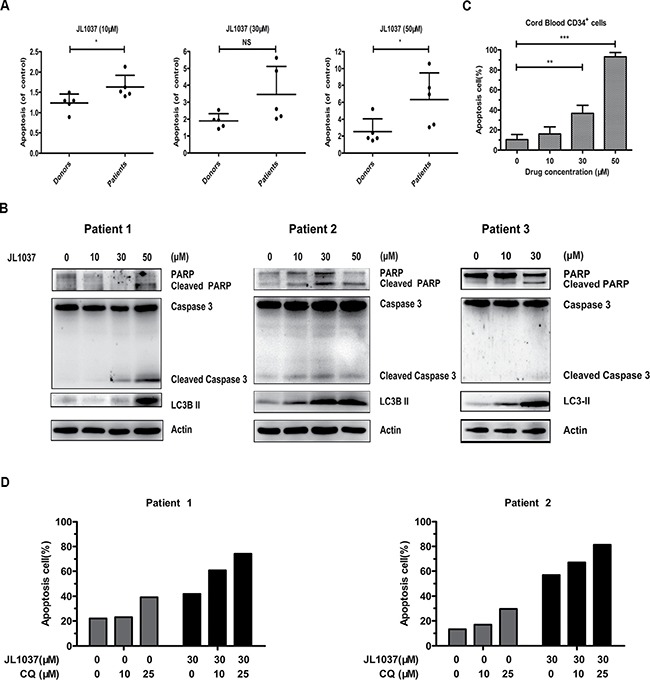
JL1037 induces cell apoptosis and autophagy in primary AML cells (**A**) BMMNCs from 5 AML patients and 5 healthy donors were seeded at a density of 5 × 10^5^ cells/ml and treated with increasing doses (0 μM, 10 μM, 30 μM and 50 μM) of JL1037 for 48 h, then the percentage of apoptotic cells was determined by flow cytometry and normalized to untreated group. (**B**) Expression of apoptotic markers (cleaved PARP, cleaved caspase 3) and autophagy-related protein (LC3-II) were measured by Western blot in AML primary cells after treated with different doses of JL1037 for 48 h. (**C**) Cord blood CD34^+^ cells were obtained through an immunomagnetic bead cell sorting system from fresh fetal umbilical cord blood samples and treated with indicated concentrations of JL1037 for 48 h. Then apoptosis analysis was performed by flow cytometry after Annexin V/PI staining. (**D**) Apoptosis analysis of AML primary cells treated with JL1037 (30 μM) in the absence or presence of autophagy inhibitor CQ (10 μM, 25 μM ) for 48 h.

We further examined the effects of JL1037 on human primary AML cells. BMMNCs from newly diagnosed AML patients were collected and treated with increasing doses of JL1037 for 48 hours. Apoptosis assay was then performed and the relative apoptosis rate was used to normalize apoptosis between different individuals. BMMNCs from healthy donors were also analyzed to evaluate the JL1037 specific killing effect on AML cells. As shown in Figure 6A, the proportion of apoptotic cells increased with the concentration of JL1037 in AML samples, while BMMNCs from healthy donors were slightly affected. Apoptosis of primary AML cells was confirmed by Western blot analysis of cleaved caspase 3 and cleaved PARP after the treatment with JL1037 for 48 hours (Figure 6B). The effects of JL1037 on CD34^+^ hematopoietic stem/progenitor cells were also examined. CD34^+^ cells were isolated from fresh umbilical cord blood samples with immunomagnetic beads and treated with different concentrations of JL1037 for 48 h, the percentage of apoptotic cells was determined with flow cytometry. The results showed that JL1037 induced apoptosis of CD34^+^ cells evidently at the high concentration of 50 μM, suggesting JL1037 at high concentrations might have toxic effect on hematopoietic cells (Figure 6C). Next, the role of autophagy response induced by JL1037 was also evaluated on primary AML cells. Treatment of AML cells with JL1037 in the presence or absence of autophagy inhibitor chloroquine (CQ) demonstrated that combination of the two compounds had better inhibitory effect than either of the single compound alone (Figure 6D).

### JL1037 prolongs survival of AML mice

**Figure 7:**
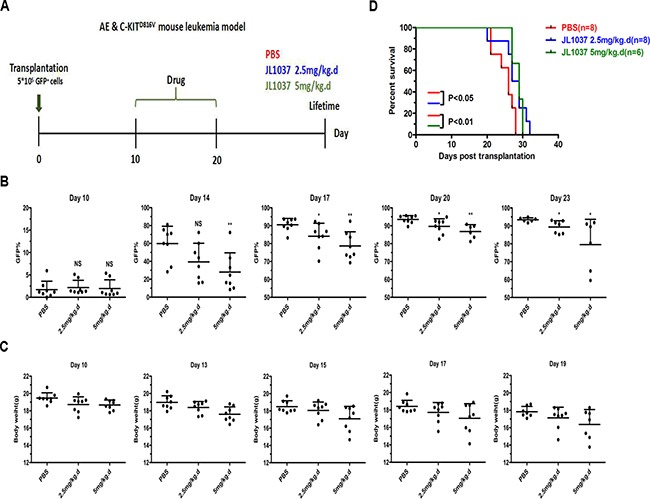
JL1037 prolongs survival of leukemia mice (**A**) *In vivo* therapeutic strategy of JL1037 on AE & C-KIT murine leukemia model. (**B**) Dynamic monitoring peripheral GFP+ cells in each group by flow cytometry. The *P*-values were determined using Student's *t*-test, *P < 0.05, and **P < 0.01. (**C**) Weight changes of mice in different treatment groups were recorded during the administration of JL1037. (**D**) Kaplan-Meier survival curves of different groups. The *P*-values were determined by log-rank test.

To further evaluate the *in vivo* anti-leukemia effects of JL1037, the survival time of the transplantable leukemia mouse model co-expressing AML1-ETO and HyC-KIT^D816V^ was investigated. Recipient mice were exposed to sublethal dose of radiation and transplanted with 5 × 10^5^ GFP^+^ spleen cells to regenerate leukemia. On Day 10 after transplantation, recipient mice were divided randomly into three groups receiving 2.5 or 5 mg/kg JL1037 or PBS for 10 days, respectively (Figure 7A). The percentages of GFP^+^ cells in peripheral blood were dynamically monitored after treatment and they were found much lower in JL1037 treated groups than that in PBS treated group at each time point (Figure 7B). Moreover, treatment with JL1037 prolonged survival time of leukemia mice, as compared with the treatment with PBS (2.5 mg/kg, *p* < 0.05; 5 mg/kg, *p* < 0.01), except for two mice in high-dose JL1037 group (5 mg/kg) died as the result of drug toxicity (Figure 7D).The median survival time of PBS group, 2.5 mg/kg and 5 mg/kg JL1037 group was 26, 27, 29 days, respectively. We monitored the changes of mouse body weight during the study to assess any side effects of JL1037 on the general conditions of the tested mice. A slight degree of weight loss was observed in both dosages of JL1037 treated groups, but not in the PBS treated control group (Figure 7C), which suggested JL1037 might also have some toxic effects along with anti-leukemia effects *in vivo*.

## DISCUSSION

High LSD1 expression correlates with poor prognosis in various kinds of malignancies [13, 24] and has been demonstrated an essential regulator of LSC. Inhibition of LSD1 abrogates clonogenic potential and induces differentiation of both murine and primary human MLL leukemia cells [17]. Despite the progresses made in recent years in AML treatment with chemotherapy and targeted therapeutics, the survival rate of AML patients remains far from satisfactory, the high recurrence rate of AML patients presents a huge challenge and unmet medical need, successfully targeting LSD1 with small molecules will no doubt provide a novel approach for treating AML patients, mainly by preventing the recurrence of AML patients due to more effective control of LSCs.

Up to date, rapid progresses have been made in the development of irreversible LSD1 inhibitors with at least 2 compounds entering early clinical stage, whereas, the development of reversible inhibitors lags behind largely due to lack of a clear structure–activity relationship (SAR) and a small number of highly selective and potent compounds. In the present study, we disclosed a novel, potent LSD1 inhibitor JL1037 and evaluated the therapeutic effect of JL1037 on AML with both *in vitro* and *in vivo* experiments. JL1037 was identified from computational screening and designed using the crystal structure of the LSD1 in complex with CoREST and a substrate-like peptide as docking template (Figure 2A). JL1037 docked well into the active site of LSD1 both spatially and chemically through non-covalent intermolecular interaction, suggesting it is a reversible inhibitor against LSD1 (Figure 2B). In addition, JL1037 specifically targeted LSD1 without significantly inhibiting MAO-A and MAO-B, showing its excellent target selectivity (Figure 2C, Table 1). In cellular experiments, JL1037 treatment resulted in an accumulation of H3K4Me1 and H3K4Me2 modifications (Figure 2D), which suggested it could effectively target LSD1 in AML cells. However, the IC_50_ value of JL1037 in cellular experiments was 200~300-fold higher than that in cell-free enzyme assay (Figure 2C, Table 1, Figure 3A). This was possibly due to the limited cell membrane permeability of this compound and the complex regulation of LSD1 activity in intracellular environment [25]. Other associated factors such as CoREST and HDACs may influence the activity of LSD1 to demethylate nucleosomal substrates.

LSD1 inhibitors are effective in a panel of leukemia cells, especially those harboring MLL-AF9 and RUNX1-RUNX1T1 translocations [17, 26]. THP-1 and Kasumi-1 cells with high LSD1 expression relative to normal BMMNCs were selected for the evaluation of JL1037 as an anti-leukemic agent (Figure 1). JL1037 reduced cell viability of THP-1 and Kasumi-1 cells through blocking cell cycle and inducing apoptosis. The likely mechanism could be explained by the upregulation of CDK inhibitors, P21 and P57 (Figure 4A), which negatively regulated cell cycle progression through inhibiting the activity of cyclin/CDK complexes [27]. The apoptotic effect of JL1037 were probably through mitochondrial death pathway, as revealed by an increase of pro-apoptotic protein Bax and a decrease of anti-apoptotic proteins Bcl-2, Bcl-XL (Figure 4C) [28]. Morphological changes such as mitochondrial swelling and degeneration were consistent with the above findings (Figure 5A). Interestingly, the efficacy of JL1037 toward AML cells was not seen with normal BMMNCs from healthy donors (Figure 6A). However, CD34^+^ hematopoietic stem/progenitor cell was not spared by the inhibition of JL1037 due to its high expression of LSD1 as shown in Figure 6C. This finding further demonstrates LSD1 plays essential roles in stem cell maintenance and is consistent with previously reported myelosuppression caused by LSD1 inhibitors [17]. Therefore, side effects such as anemia and thrombocytopenia warrant close monitoring during the early development and later on human trials of LSD1 inhibitors.

Autophagy, a catabolic process which helps cells to maintain normal homeostasis and survive against cellular stress, can be induced by many chemotherapeutic drugs in leukemia treatment, such as daunorubicin, dasatinib, HDACi, As_2_O_3_ and ATRA [21–23, 29, 30]. In present study, we first reported that autophagy could be activated by a LSD1 inhibitor in AML cells. We found that treatment with JL1037 could induce dose-dependent autophagy responses in AML cells (Figure 5). However, the exact role of autophagy in anti-leukemic therapy remains controversial. On one hand, autophagy induced by chemotherapeutic drugs are cytoprotective and associated with chemoresistance by activation of ERK in myeloid leukemia cells [22, 23]. On the other hand, autophagy induced by ATRA and/or As_2_O_3_ contributes significantly to the degradation of oncoproteins such as PML/RARα and BCR-ABL and the regulation of therapy-induced differentiation in the case of acute promyelocytic leukemia [30–33]. In this study, we found that JL1037 induced autophagy even at lower dosages than that for cell apoptosis (Figures 3C, 4B, 5B), autophagy inhibitor CQ enhanced the apoptotic effect of JL1037 (Figure 5D, 5E). As CQ alone was seen apoptotic itself in our study (Figure 5D, 5E), it remains unclear whether the enhanced apoptotic effect of JL1037 was a synergy or a forced increase due to the specific block of autophagy by CQ. Similar results were seen with primary leukemic samples directly obtained from leukemic patients (Figure 6D). Nevertheless, the observation of the enhanced apoptotic effect of JL1037 by CQ indicates that combination treatment with an autophagy inhibitor will improve the anti-leukemic efficacy of LSD1 inhibitors. Due to the low bioavailability (5%, data not shown) of JL1037, once daily intravenous injection of the compound was applied for the *in vivo* efficacy tests in a murine leukemia model co-expressing AML1-ETO and HyC-KIT^D816V^. JL1037 significantly suppressed the proliferation of the peripheral leukemia cells and slightly prolonged the survival time of leukemia mice compared with PBS treated control mice (Figure 7B, 7D). The relatively low efficacy observed in the animal study vs the strong effects observed in the cell study could probably be explained by short half-life of JL1037 (1 hour, data not shown) and the dose limiting toxicities. Body weight loss in several mice in high dose group and two animal deaths were observed in the same group (Figure 7C, 7D). The nature and extent of the toxicity were not yet fully investigated at this stage of development. Further optimization of the compound series is warranted in terms of the improvement of metabolic stability, toxicity profile and the *in vivo* efficacy.

In summary, the novel LSD1 inhibitor JL1037 exhibited strongly anti-leukemia effect on AML cell lines and primary AML cells, and also significantly prolonged survival time of leukemia mouse harboring AML1-ETO translocation. Co-treatment with an autophagy inhibitor could achieve superior apoptotic effect than JL1037 alone. JL1037 represents a promising chemical series, out of which more successful investigational drug candidates may be developed.

## MATERIALS AND METHODS

### Ethics statement

Investigations have been conducted in accordance with the approved guidelines and the entire experimental protocols were approved by the Institutional Animal Care and Use Committee of Peking Union Medical College.

### Reagents

Compound JL1037 was supplied by Fujian Jinler Pharmaceuticals and was dissolved in PBS. Chloroquine (CQ) was purchased from Sigma-Aldrich (St. Louis, MO, USA) and also dissolved in PBS.

### Cells and culture condition

AML cell lines (NB4, HL-60, U937, K562, THP-1, Kasumi-1) were cultured in RPMI 1640 containing 10% ~ 20% FBS at 37°C in 5% CO_2_. Bone marrow samples were obtained from 5 AML patients and 5 health donors enrolled in the Institute of Hematology and Blood Diseases Hospital, Chinese Academy of Medical Sciences and Peking Union Medical College. Bone marrow mononuclear cells (BMMNCs) were isolated by density gradient centrifugation using Ficoll solution (TBD Science, China) and then cultured in RPMI 1640 containing 10% FBS at 37°C in 5% CO_2_. Umbilical cord blood CD34^+^ cells were obtained through an immunomagnetic bead cell sorting system from fresh fetal umbilical cord blood samples and were cultured in IMDM supplemented with 10% FBS, 100 ng/ml SCF, 50 ng/ml TPO, and 100 ng/ml Flt-3L at 37°C in 5% CO_2_. All human samples used in experiment were collected under informed consent from the participating subjects.

### MTS assay

Cell growth inhibitory rates were measured by using CellTiter 96^®^ AQ_ueous_ One Solution Assay (Promega, USA). Cells were seeded in 96-well plates (2 × 10^4^ cells/well) in 100 μl volume and treated with various concentrations of JL1037 for 24, 48 and 72 hours. At the end of culture, 20 μl MTS reagent was added. Then the cells were incubated at 37°C for an additional 2 h and the absorbance was measured by Synergy H4 Hybrid Microplate Reader (Biotek, USA) at the wavelength of 490 nm.

### Cell cycle analysis

Cells were treated with indicated concentrations of JL1037 for 24 h at a density of 5 × 10^5^ cells/ml and then fixed with 70% ethanol overnight at 4°C. Then cells were incubated with 100 μg/ml RNase (TIANGEN, China) for 15 min and stained with 50 μg/ml PI (Sigma, USA) for 15 min at room temperature in darkness. The DNA content was analyzed with flow cytometry (LSRII, BD, USA) and cell cycle distribution was calculated by the ModFit software.

### Apoptosis assessment by Annexin V staining and morphological analysis

Cells were treated with indicated concentrations of JL1037 or CQ for 48 h at a density of 5 × 10^5^ cells/ml. Then cells were collected and stained with Annexin V-Alexa Fluor 647-A and PI (BioLegend, USA) according to the manufacturer's instructions. Apoptosis assay was performed with flow cytometry (LSRII, BD, USA) and Annexin V-positive cells were identified as apoptotic cells. The cytospins of treated cells were prepared and stained with Wright-Giemsa solution. The morphological images were captured using a Nikon Eclipse 50i microscope (Nikon Inc., Melville, NY, USA).

### LSD1 activity assay

LSD1 enzyme inhibition assay was performed with LSD1 Fluorimetric Drug Discovery kit (# BML-AK544, Enzo Life Science Inc, USA) modified according to the manufacturer's protocol. Briefly, in each well of a 384-well microplate, add 15 μl Mix A containing 1.5 μl LSD1 (0.1 μg/μl) and 0.5 μl HRP stock solutions and 13 μl assay buffer. Then, add in each well 1 μl of serial diluted compound solutions such as JL1037 (Each compound was serial diluted in 8 different concentrations in assay buffer from 0 to 10 μM) and incubate at room temperature for 5 min. Add in each well 15 μl of Mix B containing 1.2 μl of H3K4me2 (0.5 mM) and 0.4 μl of Cellestial^®^ (100X) stock solutions and 13.4 μl LSD1 assay buffer. Tap the microplate and read immediately the fluorescent signals in kinetic mode of the samples in Beckman Coulter DTX 880 Multimode Detector (535 nm and 595 nm for excitation and emission wavelengths, respectively).The assay results were plotted with Prism GraphPad.

### MAO-A and MAO-B inhibition assay

MAO-A and MAO-B enzymes were purchased from Sigma-Aldrich (St. Louis, MO, USA) and the inhibition assay was tested with Promega MAO-Glo™ Assay kit (# V1401, Madison, WI, USA) according to the manufacturer's protocol. The luminescent signals were detected with Beckman Coulter DTX 880 Multimode Detector and the results were analyzed with Prism GraphPad.

### RNA isolation and real-time quantitative PCR (qRT-PCR)

Total RNA was extracted from 5 × 10^6^ cells using RNAiso Plus (Takara, Japan) and 2 μg RNA was reverse-transcribed into cDNA using M-MLV Reverse Transcriptase (Life technologies, USA). qRT-PCR analysis for the expression of LSD1 was performed on the ABI PRISM 7500real-time PCR system with SYBR Green PCR kit (Takara, Japan) following the manufacturer's instructions. Primers for LSD1 were 5′-GCTCGGGGCTCTTATTCCTA-3′ (forward) and 5′-CCCAAAAACTGGTCTGCAAT-3′ (reverse). Human GAPDH was used as an internal control.

### Western blot assay

Following the drug treatments, cells were collected and resuspended in RIPA lysis buffer (Beyotime, China) and lysed by ultrasonic instrument. Then equal protein amount of the cell extracts was analyzed on a 12% SDS-polyacrylamide gels and transferred onto PVDF membranes. Membranes were blotted with primary antibodies targeting the following proteins: LSD1 (Sangon Biotech, Shanghai, China), pan-acetyl-H3 (Millipore, USA), Mono-Methyl-Histone H3 (Lys4), Di-Methyl-Histone H3 (Lys4), Tri-Methyl-Histone H3 (Lys4), Mono-Methyl-Histone H3 (Lys9), Di-Methyl-Histone H3 (Lys9), Tri-Methyl-Histone H3 (Lys9), PARP [poly(ADP-ribose) polymerase], Caspases-3, P21, P27, P57, Bcl-xL, Bcl-2, Bax and LC3B (all from Cell Signaling Technology, USA). β-actin (Abcam, USA) and Histone H3 (Abcam, USA) were used as internal controls. Then, membranes were incubated in HRP-conjugated secondary antibody and visualized using the HRP Substrate (Millipore, USA) by Image Quant LAS-4010 system (GE Healthcare, USA).

### Immunofluorescence analysis

Treated cells were fixed with 4% paraformaldehyde for 15 min, permeabilized with 0.25% Triton X-100 for 10 min and blocked with PBS containing 2% goat serum for 30 min at room temperature. Cells were then incubated with an anti-LC3B primary antibody (Cell Signaling Technology, USA) overnight at 4°C, washed three times with PBS and stained with an Alexa 488-labeled donkey anti-mouse IgG secondary antibody (BioLegend, USA ) 2 h at room temperature in darkness. Nuclei were stained with DAPI for visualization. Fluorescence images were taken on a spinning disk confocal microscope using a 100X oil-immersion objective. Autophagy was evaluated by the formation of punctate fluorescent structures.

### Transmission electron microscopy (TEM) assay

After the treatment with or without JL1037 for indicated hours, cells were fixed with 2.5% glutaraldehyde, post-fixed in 1% osmium tetroxide and dehydrated by a series of ethanol washes before being embedded in Epon812. Ultra-thin sections were obtained and observed by a JEM-2100F transmission electron microscope.

### *In vivo* studies using an AML1-ETO & C-KIT leukemia mouse model

The AML1-ETO & C-KIT leukemia mouse model was previously established by our laboratory [34]. For *in vivo* study, 6~8 week female C57BL/6 mice were exposed to sub-lethal of radiation (450 cGy) and transplanted with 5 × 10^5^ GFP^+^ spleen cells from the third generation leukemia mouse. The mice were monitored for 10 days and then randomly divided into three groups: PBS group, low-dose JL1037 (2.5 mg/kg.d) group and high-dose JL1037 (5 mg/kg.d) group. Each group included 8 mice. The intraperitoneal administrations of drugs were initiated on day 10 after transplantation. During a 10-day administration period, the percentage of GFP^+^ cells in peripheral blood was measured by flow cytometry every three days to assess the development of leukemia. Body weight was measured every two days to evaluate drug toxicity. The overall survival of each group was calculated from the date of transplantation to the date of death. All animal experiments were performed under protocols approved by the Institutional Animal Care and Use Committee of Peking Union Medical College.

### Statistical analysis

All experiments in this study were conducted at least three times. IC_50_ values of MTS assay was calculated using SPSS software (version 16.0). The comparisons were performed by Student's *t*-test analysis using GraphPad Prism (version 5.0). The lifespan of mice was analyzed by Kaplan-Meier methods and a log-rank test. *P*-values < 0.05 were considered statistically significant.

This work was supported by National Natural Science Foundation of China (81430004, 81570147) and CAMS Initiative Fund for Medical Sciences (2016-I2M-1-001).
